# Obstructive sleep apnea and hypopnea syndrome: cephalometric analysis

**DOI:** 10.1016/S1808-8694(15)31338-0

**Published:** 2015-10-20

**Authors:** Cristina Salles, Paulo Sérgio Flores Campos, Nilvano Alves de Andrade, Carla Daltro

**Affiliations:** ^1^ Preceptor, Medical Residence Program in Otorhinolaryngology, Santa Casa de Misericórdia da Bahia, Specialist in Otorhinolaryngology and Sleep Medicine; ^2^ Ph.D. in Oral Diagnosis (Sub-area of Radiology), Joint Professor, School of Dental Sciences, Federal University of Bahia; ^3^ Master, Federal University of Bahia. Ph.D. in Otorhinolaryngology, USP. Head of the Residence Program in Otorhinolaryngology and Head and Neck Surgery, Santa Casa de Misericórdia da Bahia (Hospital Santa Izabel); ^4^ Endocrinologist. Master, Federal University of Bahia. Professor, Escola Bahiana de Medicina e Saúde Pública. Unit of Otorhinolaryngology, Santa Casa de Misericórdia da Bahia (Hospital Santa Izabel)

**Keywords:** obstructive sleep apnea, sleep apnea syndrome, cephalometric analysis

## Abstract

**O**bstructive sleep apnea and hypopnea syndrome (OSAHS) are characterized by repeated pauses in breathing during sleep, usually associated with sleep interruption and decreased oxyhemoglobin saturation. Cephalometric analysis has become an important method in diagnosis, reporting specific craniofacial characteristics such as posterior air pharyngeal space, tongue length and hyoid position, which may predispose some people to develop SAHOS. The purpose of this revision is to present several anatomic aspects by cephalometric analysis that may have a predisposition to the development of upper airway occlusion.

## INTRODUCTION

Obstructive sleep apnea-hypopnea syndrome (OSAHS) are characterized by repeated episodes of obstruction of the upper airways during sleep, usually associated with sleep interruption and decreased oxyhemoglobin saturation^1^. According to Young et al.^2^, frequency of OSAHS in the middle-age population is of 4% among men and 2% among women. However, according to American Academy of Pediatrics^3^, prevalence of OSAHS in children may range between 0.7% and 10.3%, with no gender predominance.

Diagnosis of OSAHS is confirmed by polysomnography, where apnea in adults is considered a pause in breathing for 10 seconds or more, and hypopnea is considered a 50% reduction of the air flow for a period equal to or higher than 10 seconds, associated with over 3% decrease of oxyhemoglobin saturation and/or a minor awake. A person is classified as apneic when he/she presents 5 or more respiratory events (apneas and/or hypoapneas) per hour of sleep^1^. However, regarding children and according to the American Thoracic Society^5^, it is a consensus that the polysomnographic findings are normal when there is less than 1 respiratory event of apnea- hypoapnea per hour, with minimal duration of less than 5 seconds, oxyhemoglobin saturation over 90% and carbon dioxide at the end of exhaling below 10% of the total sleeping time^6^.

Surgical treatment has not shown to be effective, although it has improved along with the development of etiological diagnostic techniques, that is, the identification of the obstructive point of the airways. Several methods have been used with this objective, among which we have cephalometry^7^.

Cephalometric radiography is a technique used for the diagnosis of craniofacial deformities^8^, through which much data may be obtained, such as measures of the skull base, position of the hyoid bone, mandible configuration, posterior air space of the pharynx, tongue dimensions, uvula width and length, among other measures^9^. Anatomical changes at these sites may predispose the patient to obstructive sleep apnea-hypopnea syndrome.

### Cephalometric Analysis in the Etiological Diagnosis of OSAHS

Rivlin et al.^10^ have verified through cephalometric analysis that the pharyngeal transversal area in apnea patients is nearly 3.7 cm^2^, whereas it is 5.3 cm^2^ in healthy individuals. Length of the soft palate is 48mm in individuals with apnea, whereas it is around 35mm in healthy people. This marked increase of the soft palate leads to reduction of the nasopharynx and increased contact between the soft palate and tongue, leading to a collapse in this area^8^.

Skeleton measures have a significant role in the dimensions of upper airways (UA). The main parameters of pharynx are the posterior air space, that is, the distance between the pharynx posterior wall and base of tongue or soft palate. This region may be divided among three different levels: upper posterior air space (between pharynx posterior wall and the soft palate posterior line, at palatine plane level); middle (between pharynx posterior wall and inferior limit of uvula); and inferior (between pharynx posterior wall and base of tongue, at mandible line level). Air space reduction is evident among OSAHS patients, especially at the uvula level and mandible plane^11^. The most important finding obtained with cephalometry in apnea patients is the reduction of the velopharyngeal space (posterior air space), which is frequent in 86% of the cases^9^. However, Powell et al.^12^ observed that 75% of the patients presented more than one obstructive site.

Position of the hyoid bone is significantly important. In healthy patients, the hyoid bone is found at the C3-C4 cervical vertebrae, while in OSAHS patients it is usually at C4-C6 level^5^. Distance between the hyoid bone and mandible plane tends to be greater (27.8mm) in patients with OSAHS than in healthy individuals (12mm)^13^.

Nearly 58% of apnea patients presented mandibular micrognathism and retrognathism in relation to the maxilla^14^. In normal conditions, inhaling produces a flow characterized by increased muscular activities of the pharyngeal dilators (more than 20 muscles) to minimize narrowing caused by negative intraluminal pressure. Out of those, genioglossus, the tongue's muscle, is the most investigated structure as it plays an important role in pharynx dilatation^15^. As the genioglossus muscle is known to be inserted in the mandible, it is expected that a small or retrognathia mandible will approximate base of tongue and pharyngeal posterior wall, resulting in reduction of oropharyngeal dimensions. Moreover, it is believed that the phasic activity of the genioglossus muscle during breathing is reduced in OSAHS patients and that posterior tongue displacement may occur, leading to UA occlusion^10^. Conversely, Fogel et al.^16^ demonstrated that genioglossus muscular activity is higher in OSAHS patients due to negative pressure of the UA when compared to healthy individuals, which means smaller pharynx and the need for progressively higher intrapharyngeal pressure to reach an adequate airway patency. Consequently, the increased negative pressure would increment the phasic activity of the genioglossus. In addition, it is known that this muscle receives direct medullar signals (hypoglossal motor system) and may also be influenced by voluntary stimuli and the action of peripheral and central chemoreceptors (PaO_2_ and PaCO_2_). The hypoglossal motor system is stimulated by adrenergic, serotoninergic and cholinergic neurotransmitters via hypoglossal nucleus situated in the central nervous system^15^.

Endo et al.^17^ observed through cephalometric analysis that the tongue tends to occupy more UA space in obese patients when compared to non-obese OSAHS patients, both vertically and horizontally. Reduction of the air space may be explained by fat deposition on the tongue of these patients^11^.

The most common cause of OSAHS in children is adenotonsillar hypertrophy^18^, which leads to narrowing of the rhinopharyngeal space and, by consequence, to mouth breathing (96.2%). Cephalometry may allow observation of significant enlargement of the jaw and of intermaxillary structures, indicating posterior rotation of the mandible and significant retroposition of the mandible plane which may result in subsequent vertical facial growth – a typical mouth breather trace. Another finding that should be considered is the reduction of maxillary and/or mandibular growth^19^. Children with OSAHS may also present small jaw and high hard palate^20^. Those anatomical characteristics are all revealed by cephalometry.

Attention must be paid to the fact that children submitted to adenotonsillectomy as treatment for OSAHS may present snoring relapse during teenager years. This fact may be related to the consequences of craniofacial changes, probably due to early onset of VAS obstruction^21^. Facial growth is almost completed between the ages of 15 and 16 years in girls and between 18 and 19 years in boys. However, the greater growth increment occurs during infant ages. In fact, near the age of 4, this growth will have reached around 60% of its adult size, and 90% of adult dimensions at the age of 12^22^.

The collapse mechanism of UA in men and women is different and is related with local anatomical differences^23^. One finding regards the angle between the hard palate and the soft palate, that is, men present higher angle (47.1º) than women (43.3º)^24^. Moreover, Malhora et al.^25^ have verified that, as the pharyngeal pressure becomes progressively negative, the uvula and tongue are positioned against the posterior pharyngeal wall. However, at each negative value, UA collapse is smaller in women than in men. In fact, pharyngeal pressure of -13cmH_2_O resulted in UA collapse in men, while in women it represented a patent UA. UA collapse occurred in women only under a pressure of -18 cmH_2_O.

Non-obese OSAHS patients tend to present the following anatomical craniofacial characteristics: caudal hyoid, increased soft palate dimensions and consequent anteroposterior reductions of the airways at the soft palate level, reduction of anteroposterior region of nasopharynx and oral pharynx. Obese OSAHS patients presented the above findings plus increased volume of tongue and anterior hyoid bone. Lower and anterior position of hyoid bone in obese patients seems to be related to increased fat deposition on the tongue, which increases its volume.^26^

OSAHS patients presenting maxilla and mandible within normal limits, that is, 96mm and 125mm, respectively, may reveal a retropositioned mandible as well as caudal hyoid bone^27^.

Cakirer et al.^28^, using two anthropometric measurements - the cranial index (the ratio between the highest cranial width and the highest cranial length, that is, highest width x 100/highest length) and the facial index (the ratio between the nasogenian height and the bizygomatic width, that is, the nasogenian height X 100/bizigomatic length), could determine the patient's facial type. They observed that Caucasian patients with OSAHS were increasingly prone to have the brachiocephalic type of face (cranial shape associated with reduced anteroposterior dimensions), while African-Americans with OSAHS tended to have the dolichocephalic type of face, which is a long face with increased craniofacial dimensions. The brachiocephalic cranial shape resulted in small anteroposterior dimensions of the skull base and reduced anteroposterior dimensions of the VAS. Therefore, this group presented higher risk of collapsed UA. On the contrary, there are certain situations where OSAHS patients have dolicocephalic characteristics (vertical type of face) with dorsocaudal-rotated mandible leading to retroposition of tongue^11^. Redline et al.^29^, verified that young African-Americans may present higher risks for OSAHS when compared to Caucasians.

Another important feature that should be considered is that most snoring individuals with OSAHS are mouth breathers during sleep. As mouth opens 1.5cm, there is a 1cm-dorsal displacement of the mandible angle, resulting in a 1cm-reduction between the pharyngeal posterior wall and the tongue dorsum. This new tongue posture causes static and dynamic stimuli over the palatine veli, uvula, tonsillar pillars, palatine tonsils and epiglottis, leading to hypotony and hypertrophy of these structures. With muscular loosening and action of inhaling pressure during sleep, there is pharyngeal collapse and, consequently, OSAHS^30^.

## CLOSING REMARKS

The cephalometric analysis is valuable for the etiology of OSAHS and should be considered among routine exams. Cephalometry is a diagnostic procedure to collect information on skeleton abnormalities and soft tissues of patients with OSAHS^31^, providing support for indication of surgery. This should be based on disease severity and presence of anatomical alterations of UA and of craniofacial skeleton^32^.
